# Supplementary material for: Cost of childhood cancer treatment in Ethiopia
Source: PLoS One. 2023 Jun 2;18(6):e0286461. doi: 10.1371/journal.pone.0286461 (PMC10237368; doi:10.1371/journal.pone.0286461)

S1 Figure: cost aggregation at pediatric oncology unit level in TASH July 2018–July 2019

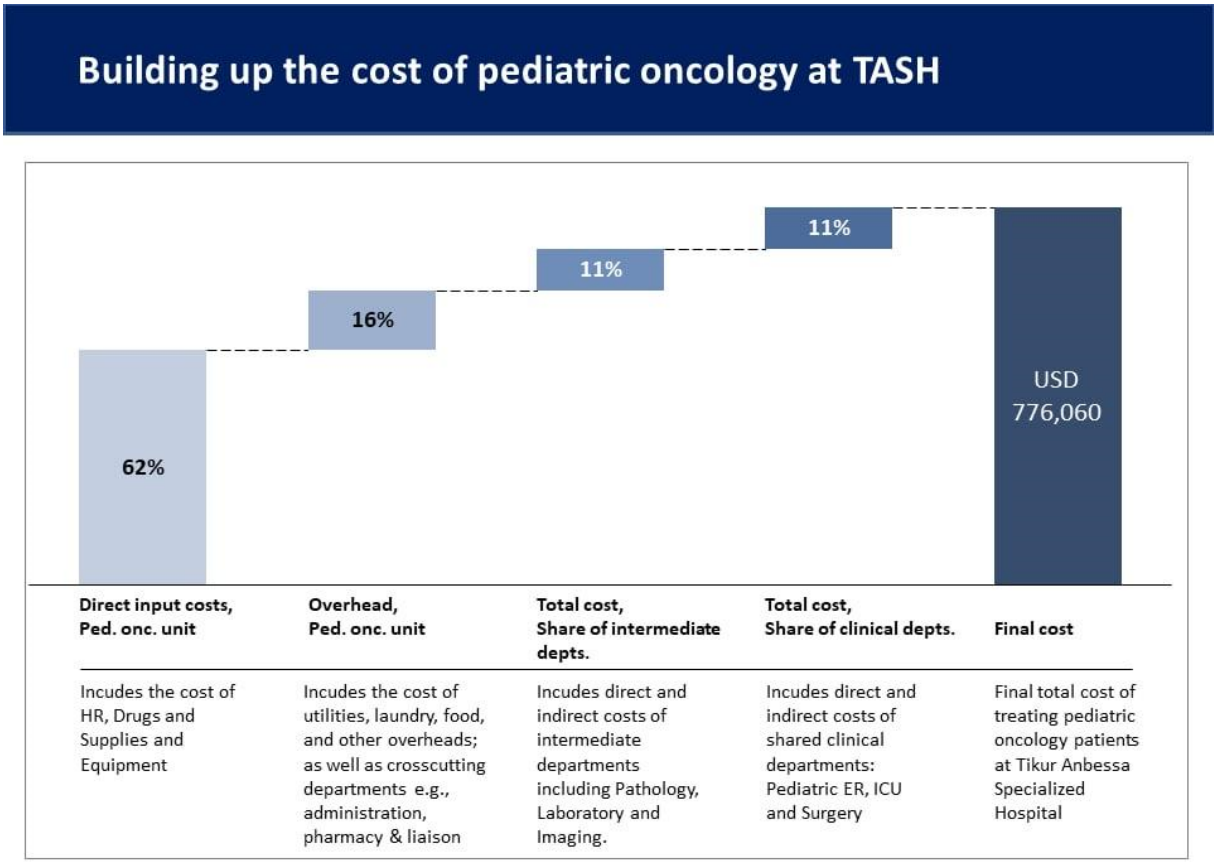

Supplement: S1 Fig — (PDF) [file pone.0286461.s001.pdf]
