# Supplementary material for: Cost of childhood cancer treatment in Ethiopia
Source: PLoS One. 2023 Jun 2;18(6):e0286461. doi: 10.1371/journal.pone.0286461 (PMC10237368; doi:10.1371/journal.pone.0286461)
Supplement: S1 Table — (DOCX) [file pone.0286461.s002.docx]

**S1Table: allocation statistics used for costing analysis**

| **Cost components** | **Allocation Base** | **Allocated to** |
| --- | --- | --- |
| ***Overhead cost*** | | |
| Utilities | M2 (AREA) |  |
|  |  | To all departments |
| Patient Food | Bed days | To IPDs |
| Laundry | Weighting factor across ER, OR IPD | To IPDs, ER and OR |
|  | [1 * # surgeries] + [(1/5) * # procedures] + [(1/10) * # bed days] + [(1/5) * 365 * # ER beds] | Tailored approach. A subset of departments was assumed to be the main consumers of laundry services (mapped by the hospital staff): Major OR, Minor OR, IPD wards, and ER. Then, the team, together with the hospital staff, estimated relative consumption of laundry for the four categories, which is given as 1 Major OR surgery = 5 Minor OR surgeries = 10 IPD bed days = 5 ER bed days. That is to say that one major surgery uses 5–10 times more laundry services |
| Other overheads (like office supplies, printing, educational supplies, fuel, per diem, training cost, etc.) | Personnel | All departments |
| ***Cross cutting departments*** | | |
| **Cost component (department)** | **Allocation Base** | **Allocated to** |
| Administrative departments (Human resources, Finance, General Service, legal etc.) cost | Personnel | All departments except Admin |
| Cost of running the Pharmacy department | Visits and Admission | OPD^a^ and IPD^b^ only |
| a) Personnel (Direct cost) |  |  |
| Cost of running the Pharmacy department | Personnel | All departments except Admin and Pharmacy |
| b) overhead cost (Indirect cost) |  |  |
| Liaison | Bed days | All IPDs |
| ***Clinical support departments*** | | |
| **Cost component (department)** | **Allocation Base** | **Allocation to** |
| Minor OR^c^ | Visits | Relevant OPDs mapped by clinicians |
| Major OR | Admission | Relevant IPDs |
| Laboratory | Patient load* | Relevant OPDs and IPDs |
|  | ∑ (All OPD visits*3, All IPD visits*1, Number of deliveries*2, All ER^d^ visits*3) | Patient load is calculated using 1Bed day = 3 OPD visits = 3 ER visits = 2Deliveries (50) |
| Radiology | Patient load | Relevant OPDs and IPDs |
| Endoscopy | Patient load | Relevant OPDs and IPDs |
| Pathology | Patient load | Relevant OPDs and IPDs |
| Triage | Visits | Relevant OPDs |
| ^a^ Outpatient department  ^b^ Inpatient department  ^c^ Operation theatre  ^d^ Emergency room | | |
