# Supplementary material for: Cost of childhood cancer treatment in Ethiopia
Source: PLoS One. 2023 Jun 2;18(6):e0286461. doi: 10.1371/journal.pone.0286461 (PMC10237368; doi:10.1371/journal.pone.0286461)
Supplement: S2 Table — (DOCX) [file pone.0286461.s003.docx]

**S2 Table: scenario-based cost sensitivity analysis in TASH July 2018- July 2019**

| ***Scenario*** | ***cost (USD)*** |
| --- | --- |
| base case (costing result from the top-down | 577 |
| low case (baseline unit cost adjusted taking incident case from the registry, 1,654) | 469 |
| high case scenario (taking cost estimate and incident number of cases (1,035) from chart review) | 1 085 |
| The estimated number of annual patients was 1,035 from the chart review and 1,654 from the department register. Since there was a problem with data quality in the pediatric oncology unit register (mainly double counting), we used the midpoint value (1,345 patients) in the base case costing analysis to account for potential underestimation in the chart review due to sampling effects and a potential overestimation of the department registry. We used the number of patients from the chart review (1,035) for the high-cost scenario and the data from the pediatric oncology unit (1,654) for the low-cost scenario estimate. | |
