# Supplementary material for: Cost of childhood cancer treatment in Ethiopia
Source: PLoS One. 2023 Jun 2;18(6):e0286461. doi: 10.1371/journal.pone.0286461 (PMC10237368; doi:10.1371/journal.pone.0286461)
Supplement: S3 Table — (DOCX) [file pone.0286461.s004.docx]

**S3 Table: cost categories share for TASH overall, adult oncology and pediatric oncology units in TASH, 2018-2019**

| Cost category | ***Cost category share*** | | |
| --- | --- | --- | --- |
|  | **Pediatric oncology** | **Adult oncology** | **TASH overall** |
| Human resource | 31% | 24% | 35% |
| Drugs & supplies | 38% | 42% | 18% |
| Equipment depreciation | 2% | 4% | 4% |
| Overhead | 18% | 16% | 15% |
| Intermediate departments | 12% | 14% | 29% |
